# Iron and Zinc Treatment in Iron Deficiency

**DOI:** 10.4274/tjh.2016.0249

**Published:** 2016-12-01

**Authors:** Beuy Joob, Viroj Wiwanitkit

**Affiliations:** 1 Sanitation 1 Medical Academic Center, Bangkok, Thailand; 2 Hainan Medical University, Hainan, China

**Keywords:** iron, Zinc, treatment, deficiency

## To the Editor,

The recent report by Özhan et al. was very interesting [1]. Özhan et al. concluded that “iron and zinc treatment instead of only iron replacement may be considered in cases of iron deficiency” [1]. The results from their study might support this suggestion. Nevertheless, we would like to add some comments. First, there was no complete nutritional evaluation in the patient and control groups, and there might have been some effects due to differences of intake among the subjects. In addition, it is not doubted that the patients had iron deficiency, but there is still the chance of the coexistence of other hemoglobin disorders. In Southeast Asia, concurrent iron deficiency and hemoglobinopathy are very common and can be misdiagnosed and incorrectly managed [2]. Iron supplementation in the case of combined iron deficiency and hemoglobinopathy has to be carefully considered [2,3]. Focusing on the serum zinc level, there is still no pathogenesis to explain the problem in the case of iron deficiency, but there is already a report confirming that hemoglobinopathy can result in low serum zinc levels [4]. Hence, to apply the recommendation of Özhan et al., further studies are required for validation, and attention to possible concomitant hemoglobinopathy is necessary [1].
