# Supplementary material for: Iron and Zinc Treatment in Iron Deficiency
Source: Turk J Haematol. 2016 Dec 1;33(4):370. doi: 10.4274/tjh.2016.0249 (PMC5204205; doi:10.4274/tjh.2016.0249)
Supplement: Related texts

# Reply: Authors' Response

**Authors:** Onur Özhan, Neslihan Erdem, İsmet Aydoğdu, Mehmet Ali Erkurt, İrfan Kuku

Dear Dr Joob,

Thank you for your comments and recommendations. We had evaluated the zinc deficiency in iron deficiency anemia, not in all anemia types. Serum iron, ferritin, and transferrin saturation levels were used in diagnosis and whether or not hemoglobinopathy exists, these patients were diagnosed iron deficiency anemia. And for possible mechanisms, there are some theories mentioned in the article. One of them is increase in production of Zn-protoporphyrin and usage of zinc instead of iron in the protoporphyrin structure [1], which can explain zinc deficiency in iron deficiency. And in another study, histopathological changes causing iron and zinc deficiency in intestinal mucosa were reversed with zinc treatment and the absorption of zinc and iron was improved [2]. But still as you mentioned and as we mentioned in our article, further studies are needed.

## References

[1] Hastka J, Lassere JJ, Schwarzbeck A, Hehlmann R (1994). Central role of zincprotoporphyrin in staging iron deficiency.. Clin Chem.

[2] Arcasoy A, Tanoshima R (1996). İnsan sağlığında çinkonun önemi. TÜBİTAK Bilim ve Teknik Dergisi.
